# Non‐pharmacological interventions for persistent postural‐perceptual dizziness (PPPD)

**DOI:** 10.1002/14651858.CD015333.pub2

**Published:** 2023-03-13

**Authors:** Katie E Webster, Tomohiko Kamo, Laura Smith, Natasha A Harrington-Benton, Owen Judd, Diego Kaski, Otto R Maarsingh, Samuel MacKeith, Jaydip Ray, Vincent A Van Vugt, Martin J Burton

**Affiliations:** Cochrane ENT, Nuffield Department of Surgical SciencesUniversity of OxfordOxfordUK; Department of Physical TherapyFaculty of Rehabilitation, Gunma Paz UniversityGunmaJapan; School of PsychologyUniversity of KentCanterburyUK; Ménière's SocietyDorkingUK; ENT DepartmentUniversity Hospitals of Derby and Burton NHS Foundation TrustDerbyUK; National Hospital for Neurology and NeurosurgeryLondonUK; Amsterdam UMC, Vrije Universiteit Amsterdam, Department of General PracticeAmsterdam Public Health Research InstituteAmsterdamNetherlands; ENT DepartmentOxford University Hospitals NHS Foundation TrustOxfordUK; University of SheffieldSheffieldUK; UK Cochrane CentreOxfordUK

**Keywords:** Adult, Humans, Chronic Disease, Dizziness, Dizziness/therapy, Republic of Korea

## Abstract

**Background:**

Persistent postural‐perceptual dizziness (PPPD) is a chronic balance disorder, which is characterised by subjective unsteadiness or dizziness that is worse on standing and with visual stimulation. The condition was only recently defined and therefore the prevalence is currently unknown. However, it is likely to include a considerable number of people with chronic balance problems. The symptoms can be debilitating and have a profound impact on quality of life. At present, little is known about the optimal way to treat this condition. A variety of medications may be used, as well as other treatments, such as vestibular rehabilitation.

**Objectives:**

To assess the benefits and harms of non‐pharmacological interventions for persistent postural‐perceptual dizziness (PPPD).

**Search methods:**

The Cochrane ENT Information Specialist searched the Cochrane ENT Register; Central Register of Controlled Trials (CENTRAL); Ovid MEDLINE*;* Ovid Embase; Web of Science; ClinicalTrials.gov; ICTRP and additional sources for published and unpublished trials. The date of the search was 21 November 2022.

**Selection criteria:**

We included randomised controlled trials (RCTs) and quasi‐RCTs in adults with PPPD, which compared any non‐pharmacological intervention with either placebo or no treatment. We excluded studies that did not use the Bárány Society criteria to diagnose PPPD, and studies that followed up participants for less than three months.

**Data collection and analysis:**

We used standard Cochrane methods. Our primary outcomes were: 1) improvement in vestibular symptoms (assessed as a dichotomous outcome ‐ improved or not improved), 2) change in vestibular symptoms (assessed as a continuous outcome, with a score on a numerical scale) and 3) serious adverse events. Our secondary outcomes were: 4) disease‐specific health‐related quality of life, 5) generic health‐related quality of life and 6) other adverse effects. We considered outcomes reported at three time points: 3 to < 6 months, 6 to ≤ 12 months and > 12 months. We planned to use GRADE to assess the certainty of evidence for each outcome.

**Main results:**

Few randomised controlled trials have been conducted to assess the efficacy of different treatments for PPPD compared to no treatment (or placebo). Of the few studies we identified, only one followed up participants for at least three months, therefore most were not eligible for inclusion in this review.

We identified one study from South Korea that compared the use of transcranial direct current stimulation to a sham procedure in 24 people with PPPD. This is a technique that involves electrical stimulation of the brain with a weak current, through electrodes that are placed onto the scalp. This study provided some information on the occurrence of adverse effects, and also on disease‐specific quality of life at three months of follow‐up. The other outcomes of interest in this review were not assessed. As this is a single, small study we cannot draw any meaningful conclusions from the numeric results.

**Authors' conclusions:**

Further work is necessary to determine whether any non‐pharmacological interventions may be effective for the treatment of PPPD and to assess whether they are associated with any potential harms. As this is a chronic disease, future trials should follow up participants for a sufficient period of time to assess whether there is a persisting impact on the severity of the disease, rather than only observing short‐term effects.

## Plain language summary

**What are the benefits and risks of treatments other than medication for persistent postural‐perceptual dizziness (PPPD)?**

**Key messages**

There are very few studies that look at treatments for persistent postural‐perceptual dizziness (PPPD). We only found one very small study, which looked at the use of electrical stimulation of the brain (called transcranial direct current stimulation).

Further work is needed in this area to help establish whether there are any treatments that may be effective at treating this condition, and to check if they cause any unwanted or harmful effects.

**What is PPPD?**

People with PPPD have symptoms of dizziness or unsteadiness, which are worse when standing up or moving around, or with rich visual stimulation (from complicated patterns or busy moving images).

**How is PPPD treated?**

Sometimes people may take medication to try and help their symptoms of PPPD. Other treatments may also be used, for example a type of physiotherapy known as vestibular rehabilitation, or talking therapies.

**What did we want to find out?**

We wanted to find out:

‐ whether there was evidence that treatments other than medication might help to improve the symptoms of PPPD;

‐ whether these treatments might cause any harm.

**What did we do?**

We searched for studies that compared different types of treatment (excluding medications) to either no treatment or placebo (dummy) treatment.

**What did we find?**

We found one small study that looked at the use of transcranial direct current stimulation for PPPD. This treatment involves applying a weak electrical current to the brain, through small electrodes that are placed on the surface of the scalp. People in the study were given a device to use at home to carry out this treatment five times per week, for a period of three weeks.

The study did assess potential harms of the treatment, and also looked at how the treatment affected dizziness‐related quality of life. However, as it was such a small study, it is unclear whether this treatment has any effect on the symptoms of PPPD, or whether there are any risks associated with treatment.

**What are the limitations of the evidence?**

Further research is needed to identify whether any treatments are useful for PPPD and whether they might be associated with any harms.

**How up‐to‐date is this evidence?**

The evidence is up‐to‐date to November 2022.

## Summary of findings

**Summary of findings 1:** Transcranial direct current stimulation compared to placebo for persistent postural‐perceptual dizziness (PPPD)

**Transcranial direct current stimulation compared to placebo for persistent postural‐perceptual dizziness (PPPD)**						
**Patient or population:** adults with PPPD **Setting:** outpatient **Intervention:** transcranial direct current stimulation **Comparison:** placebo						
**Outcomes**	Anticipated absolute effects^*^ (95% CI)		**Relative effect (95% CI)**	**№ of participants (studies)**	**Certainty of the evidence (GRADE)**	**Comments**
	**Risk with placebo**	**Risk with transcranial direct current stimulation**				
Improvement in vertigo at 3 to ≤ 6 months	No studies assessed this outcome.					
Change in vertigo at 3 to ≤ 6 months	No studies assessed this outcome.					
Serious adverse events	0/10	0/11	Not estimable	21 (1 RCT)	⊕⊝⊝⊝ **very low**^1 2^	The evidence is very uncertain about the effect of transcranial direct current stimulation on serious adverse events.

CI: confidence interval; RCT: randomised controlled trial ^1^Risk of bias and also indirectness, as a single study will not capture all techniques and methods of administration for this intervention, which may have different adverse effects. ^2^Very serious imprecision, due to the small sample size and failure to meet the optimal information size (taken as < 300 events for a dichotomous outcome).

**Summary of findings 2:** Talking therapies or stress management compared to placebo or no treatment for persistent postural‐perceptual dizziness (PPPD)

**Talking therapies or stress management compared to placebo or no treatment for persistent postural‐perceptual dizziness (PPPD)**						
**Patient or population:** adults with PPPD **Setting:** outpatient **Intervention:** talking therapies or stress management **Comparison:** placebo or no treatment						
**Outcomes**	Anticipated absolute effects^*^ (95% CI)		**Relative effect (95% CI)**	**№ of participants (studies)**	**Certainty of the evidence (GRADE)**	**Comments**
	**Risk with placebo**	**Risk with talking therapies or stress management**				
Improvement in vertigo at 3 to ≤ 6 months	No studies assessed this outcome.					
Change in vertigo at 3 to ≤ 6 months	No studies assessed this outcome.					
Serious adverse events	No studies assessed this outcome.					

CI: confidence interval

**Summary of findings 3:** Vestibular rehabilitation compared to placebo or no treatment for persistent postural‐perceptual dizziness (PPPD)

**Vestibular rehabilitation compared to placebo or no treatment for persistent postural‐perceptual dizziness (PPPD)**						
**Patient or population:** adults with PPPD **Setting:** outpatient **Intervention:** vestibular rehabilitation **Comparison:** placebo or no treatment						
**Outcomes**	Anticipated absolute effects^*^ (95% CI)		**Relative effect (95% CI)**	**№ of participants (studies)**	**Certainty of the evidence (GRADE)**	**Comments**
	**Risk with placebo**	**Risk with vestibular rehabilitation**				
Improvement in vertigo at 3 to ≤ 6 months	No studies assessed this outcome.					
Change in vertigo at 3 to ≤ 6 months	No studies assessed this outcome.					
Serious adverse events	No studies assessed this outcome.					

CI: confidence interval

## Background

### Description of the condition

Persistent postural‐perceptual dizziness (PPPD) is a chronic balance disorder that is characterised by unsteadiness or dizziness, triggered by changes in position or visual stimulation. Although the disorder was only defined in 2017, descriptions of individuals with the characteristic symptoms have been reported in the medical literature for many years (Staab 2017). The term itself has been used since at least 2013 (Staab 2020). In the past, individuals with these, or very similar, symptoms have been diagnosed with a variety of disorders, such as phobic postural vertigo, space‐motion discomfort, visual vertigo or chronic subjective dizziness (Staab 2017). PPPD includes the core features of many of these disorders. 

Criteria for diagnosis were established by expert consensus in 2017 (Staab 2017), and are based on symptoms alone. The presence of each of the following five features is required to make the diagnosis:

- dizziness, unsteadiness or non‐spinning vertigo, present on most days for at least three months;
- the symptoms are exacerbated by an upright posture, motion or exposure to complex visual stimuli;
- the disorder is triggered by an episode of unsteadiness, dizziness or vertigo ‐ caused by another balance disorder, a neurological or medical disorder, or psychological distress;
- symptoms must cause considerable distress to the sufferer;
- the symptoms should not be better accounted for by an alternative diagnosis.

As the diagnostic criteria were only recently established, accurate estimates of the prevalence and incidence of this newly characterised disorder are not yet available. However, a significant number of individuals with chronic balance problems, previously diagnosed with other conditions, may now be included within this diagnostic category. 

The pathophysiological processes underlying PPPD are poorly understood, although a model has been proposed to explain the likely mechanism (Staab 2020). This suggests that temporary changes in balance function caused by a specific event (such as an acute balance disorder, medical or psychological disturbance) become chronic, despite the resolution of the initial insult. Balance function appears to become more dependent on visual input, and individuals may be hypervigilant with regard to their own movement and balance. PPPD may reflect a maladaptation to an acute vestibular insult.

The impact of PPPD on the individual may be considerable, due to the chronic and persistent nature of the condition, and the consequences it has for day‐to‐day activities and quality of life. A small qualitative study recently identified three themes describing the impact of this disorder on individuals (Sezier 2019). These were a perception that their symptoms were not viewed as part of a valid or credible disorder, a change in their perceived self‐identity since their symptoms started, and challenges in coping with the symptoms and changes in their lives. 

### Description of the intervention

In the absence of a good understanding of the pathophysiological mechanisms underlying PPPD, it is difficult to identify potential therapies based on any *specific* mechanisms. However, a number of drugs and non‐drug interventions have been used in people with chronic vestibular symptoms (for example, chronic subjective dizziness), and these are therefore considered possible therapeutic options in people with PPPD (Axer 2020). 

Non‐pharmacological therapies include interventions that aim to manage associated psychological symptoms that are common in PPPD. These include anxiety and mood disturbance. A variety of therapies may be used, including counselling or cognitive behavioural therapy (Yu 2018). Other interventions intended to relieve stress ‐ such as meditation and mindfulness ‐ may also be used. 

Vestibular rehabilitation may also be considered. This is an exercise‐based therapy that involves walking exercises, balance retraining, and visual and postural exercises. Exercises are tailored to the individual, to account for their specific symptoms. Vestibular rehabilitation is often provided in person, on a one‐to‐one or group basis, by a therapist. However, self‐directed booklet‐based, video game‐ and internet‐based packages are now available (Choi 2021; Eldøen 2021). 

### How the intervention might work

Given the uncertainty in the pathogenesis of PPPD, at present no clear mechanism of action has been established for either pharmacological or non‐pharmacological interventions. In the current model of PPPD, anxiety is thought to promote and help perpetuate the changes in balance function that occur. Many individuals with PPPD also suffer with mood disturbance, which may be related to the underlying condition. Talking therapies or stress management strategies, which are intended to improve these symptoms, may therefore help with both the underlying disorder and with improving quality of life – by enabling people to understand and cope with their symptoms more easily.

Vestibular rehabilitation aims to retrain the balance system. Repeated exercises are used to cause habituation – a reduction in the abnormal balance signals that are being generated by routine stimuli. This allows central compensation to occur and helps to re‐establish normal balance function.

### Why it is important to do this review

Balance disorders can be difficult to diagnose and treat. There are few specific diagnostic tests, a variety of related disorders and a limited number of interventions that are known to be effective. To determine which topics within this area should be addressed with new or updated systematic reviews, we conducted a scoping and prioritisation process, involving stakeholders (https://ent.cochrane.org/balance-disorders-ent). PPPD was ranked as one of the highest priority topics during this process (along with vestibular migraine and Ménière's disease). 

The impact on quality of life, and the absence of national or international recommendations for treatment strategies, make it important to review the evidence available to manage this condition. At present, there is no guidance available for healthcare professionals and patients identifying the possible benefits or harms of different treatment options. In this review, we aim to summarise the current evidence for non‐pharmacological treatments for this condition; pharmacological therapies are addressed in another review (Webster 2022a).  

## Objectives

To assess the benefits and harms of non‐pharmacological interventions for persistent postural‐perceptual dizziness (PPPD). 

## Methods

### Criteria for considering studies for this review

#### Types of studies

We included randomised controlled trials (RCTs) and quasi‐randomised trials (where trials were designed as RCTs, but the sequence generation for allocation of treatment used methods such as alternate allocation, birth dates etc). 

If cross‐over trials were identified then these would have been included, providing the data were reported in an appropriate way to be included in the meta‐analysis. If cluster‐RCTS were identified then they would also have been eligible for inclusion, providing we could appropriately account for the clustering in the data analysis. 

We included studies reported as full‐text, those published as conference abstracts only and unpublished data. 

For studies to obtain accurate estimates of effect for different interventions, we considered that follow‐up of participants should last at least three months, as the interventions may take some time to take effect, and this is a chronic illness, whereas short‐term follow‐up may not accurately represent the longer‐term outcome for patients. Studies that followed up participants for less than three months were excluded from the review.

#### Types of participants

We included studies that recruited adult participants (aged 18 years or older) with a diagnosis of PPPD, according to the Bárány Society criteria (see Appendix 1).

We excluded from the review studies that used alternative definitions of functional dizziness syndromes, such as chronic subjective dizziness (CSD), visual vertigo, space‐motion discomfort or phobic postural vertigo. Although we recognise that the symptoms of PPPD overlap considerably with some features of these disorders, we focused the results of the review so that they are directly relevant to those who are diagnosed with this (recently characterised) condition. 

Where studies had recruited participants with a variety of diagnoses (e.g. PPPD and other distinct conditions) we planned to include the study if either:

- the majority of participants (≥ 90%) had a diagnosis of PPPD; or
- subgroup data were available that allowed us to identify data relevant specifically to those with PPPD.

However, we did not identify any studies where this was the case. 

#### Types of interventions

We planned to include the following interventions:

- talking therapies or stress management (including counselling, cognitive behavioural therapy (CBT), meditation, mindfulness);
- vestibular rehabilitation.

We considered these to be non‐pharmacological interventions that would be likely to be used for PPPD. However, due to the paucity of data available, we deviated from our protocol and included any non‐pharmacological interventions that had been used in a study which met all of our other inclusion criteria. 

The main comparisons were intended to be:

- talking therapies or stress management versus no treatment;
- vestibular rehabilitation versus no treatment.

Due to the inclusion of one study on transcranial direct current stimulation for PPPD, we added an additional comparison:

- transcranial direct current stimulation versus no treatment/placebo.

##### Concurrent treatments

There were no limits on the type of concurrent treatments used, providing these were used equally in each arm of the study. 

#### Types of outcome measures

We assessed outcomes at the following time points:

- 3 to < 6 months;
- 6 to ≤ 12 months;
- > 12 months.

The exception was for adverse event data, when we used the longest time period of follow‐up. 

We searched the COMET database for existing core outcome sets of relevance to PPPD and vertigo, but were unable to find any published core outcome sets. We therefore conducted a survey of individuals with experience of (or an interest in) balance disorders to help identify outcomes that should be prioritised. This online survey was conducted with the support of the Ménière's Society and the Migraine Trust, and included 324 participants, who provided information regarding priority outcomes. The review author team used the results of this survey to inform the choice of outcome measures in this review. 

We planned to analyse the following outcomes in the review, but we did not use them as a basis for including or excluding studies.

##### Primary outcomes

- Improvement in vestibular symptoms
  - Measured as a dichotomous outcome (improved/not improved), according to self‐report, or according to a change of a specified score (as described by the study authors) on a rating scale.
- Change in vestibular symptoms
  - Measured as a continuous outcome, to identify the extent of change in vestibular symptoms.
- Serious adverse events
  - Including any event that caused death, was life‐threatening, required hospitalisation, resulted in disability or permanent damage, or in congenital abnormality. Measured as the number of participants who experienced at least one serious adverse event during the follow‐up period.

Vestibular symptoms comprise a variety of different features, including frequency of episodes, duration of episodes and severity/intensity of the episodes. People may experience vertigo, dizziness or unsteadiness as part of this disorder. Where possible, we included data for the vestibular symptoms outcomes that encompassed all aspects (frequency, duration and severity/intensity of symptoms). 

##### Secondary outcomes

- Disease‐specific health‐related quality of life
  - Measured with the Dizziness Handicap Inventory (DHI, Jacobsen 1990), a validated measurement scale in widespread use. If data from the DHI were unavailable we planned to extract data from alternative validated measurement scales, according to the order of preference described in the list below (based on the validity of the scales for this outcome):
    - DHI short form (Tesio 1999);
    - DHI screening tool (Jacobsen 1998).
- Generic health‐related quality of life
  - Measured with a validated measurement tool that assesses global health‐related quality of life, such as the EQ‐5D‐3L (EuroQol 1990), EQ‐5D‐5L (Herdman 2011) or SF‐36 (Ware 1992).
- Other adverse effects
  - Measured as the number of participants who experience at least one episode of the specified adverse events during the follow‐up period. Including the following specified adverse effects:
    - headache;
    - gastrointestinal disturbance;
    - sleep disturbance (e.g. somnolence or insomnia);
    - psychological disturbance (e.g. anxiety, depression, agitation);
    - cardiovascular disturbance (e.g. postural lightheadedness, palpitations);
    - sexual dysfunction.

### Search methods for identification of studies

The Cochrane ENT Information Specialist conducted systematic searches for randomised controlled trials and controlled clinical trials. There were no language or publication status restrictions. We only included studies that used the definition of PPPD that was defined in 2017 (Staab 2017), and first proposed in 2013 (Staab 2020). Therefore, we restricted some of the broader search terms to a year of publication from 2010 onwards. The date of the search was 21 November 2022.

#### Electronic searches

The Information Specialist searched:

- the Cochrane ENT Trials Register (search via the Cochrane Register of Studies to 21 November 2022);
- the Cochrane Central Register of Controlled Trials (CENTRAL) (search via the Cochrane Register of Studies to 21 November 2022);
- Ovid MEDLINE(R) Epub Ahead of Print, In‐Process & Other Non‐Indexed Citations, Ovid MEDLINE(R) Daily and Ovid MEDLINE(R) (1946 to 21 November 2022);
- Ovid Embase (1974 to 21 November 2022);
- Web of Knowledge, Web of Science (1945 to 21 November 2022);
- ClinicalTrials.gov, www.clinicaltrials.gov (searched to 21 November 2022);
- World Health Organization (WHO) International Clinical Trials Registry Platform (ICTRP), https://trialsearch.who.int (searched to 21 November 2022).

The Information Specialist modelled subject strategies for databases on the search strategy designed for CENTRAL, Ovid MEDLINE and Ovid Embase. Where appropriate, they were combined with subject strategy adaptations of the highly sensitive search strategy designed by Cochrane for identifying randomised controlled trials and controlled clinical trials (as described in the Technical Supplement to Chapter 4 of the *Cochrane Handbook for Systematic Reviews of Interventions* version 6.1) (Lefebvre 2021).

#### Searching other resources

We scanned the reference lists of identified publications for additional trials and contacted trial authors where necessary. In addition, the Information Specialist searched Ovid MEDLINE to retrieve existing systematic reviews relevant to this systematic review, so that we could scan their reference lists for additional trials. The Information Specialist also ran non‐systematic searches of Google Scholar to retrieve grey literature and other sources of potential trials.

We did not perform a separate search for adverse effects. We considered adverse effects described in included studies only.

### Data collection and analysis

#### Selection of studies

At least two review authors (of KG, TK, LS and KW) independently screened the titles and abstracts using Covidence (https://www.covidence.org), to identify studies that may be relevant for this review. Any discrepancies were resolved by consensus, or by retrieving the full text of the study for further assessment. 

We obtained the full text for any study that may have been relevant and this was again checked by two authors (of KG, TK, LS and KW) independently to determine whether it met the inclusion criteria for the review. Any differences were resolved by discussion and consensus, or through recourse to a third author if necessary. 

Studies that were retrieved in full text but subsequently deemed to be inappropriate for the review (according to the inclusion/exclusion criteria) were listed as excluded studies, according to the main reason for exclusion. 

The unit of interest for the review is the study, therefore multiple papers or reports of a single study are grouped together under a single reference identification. We recorded the study selection process in sufficient detail to complete a PRISMA flow diagram (Figure 1) and the Characteristics of excluded studies table. 

**1:**
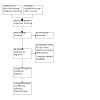
Flow chart of study retrieval and selection.

##### Screening eligible studies for trustworthiness

We assessed studies meeting our inclusion criteria for trustworthiness using a screening tool developed by Cochrane Pregnancy and Childbirth. This tool includes specified criteria to identify studies that are considered sufficiently trustworthy to be included in the review (see Appendix 2). If any studies were assessed as being potentially 'high risk', we contacted the study authors to obtain further information or address any concerns. We planned to include the data from any studies with persisting concerns only with a sensitivity analysis (see Sensitivity analysis). The process is outlined in Figure 2. 

**2:**
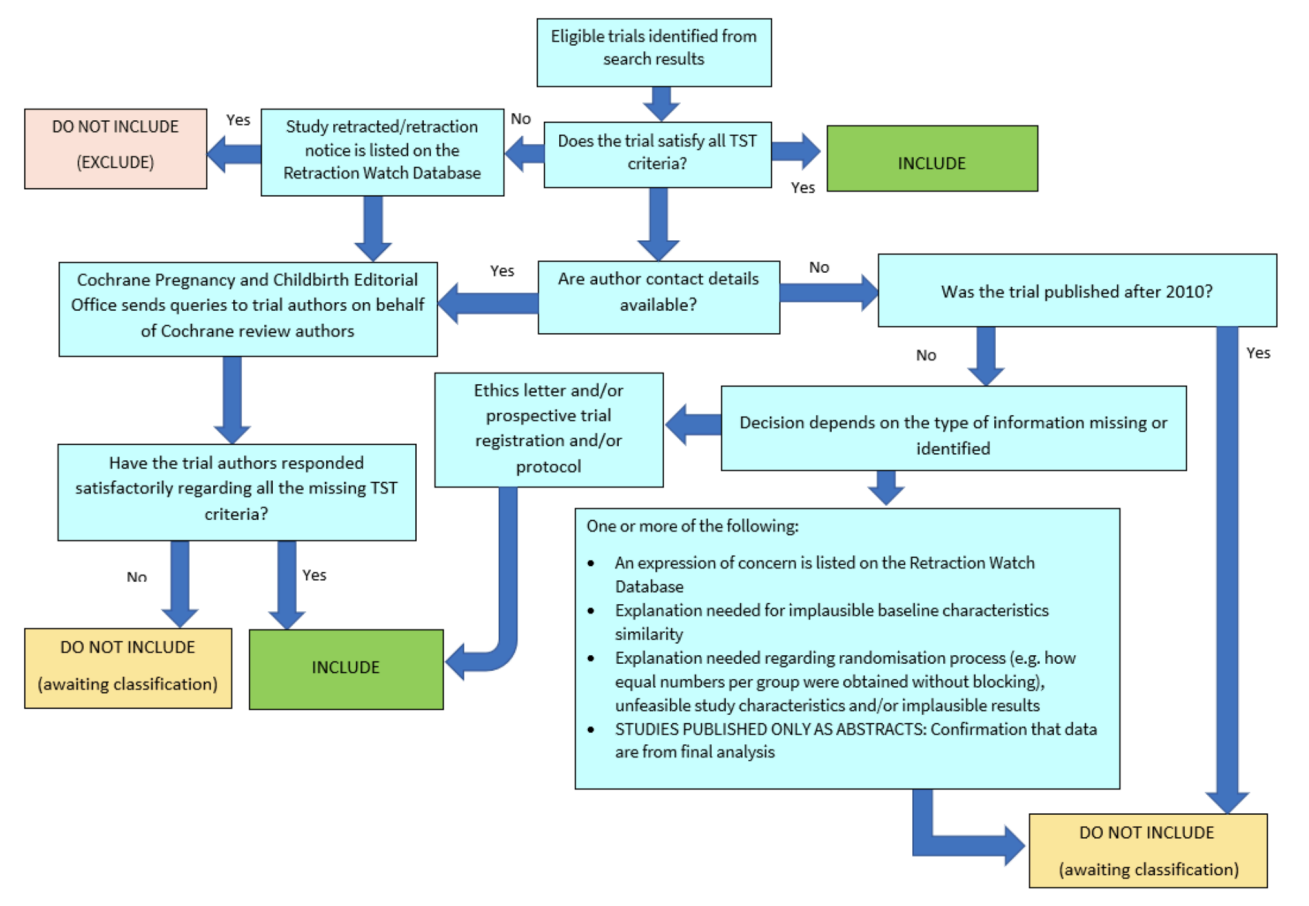
The Cochrane Pregnancy and Childbirth Trustworthiness Screening Tool

However, we identified only one study for inclusion in this review (Im 2022). We did have some concerns about the randomisation process for this study, as identical numbers of participants were allocated to each group, and there was no report of blocked randomisation. We contacted the authors, who stated that "The number of each group was just a coincidence". We had no other concerns when using the trustworthiness screening tool, therefore we have included this study in the review. However, we note that there may be some concerns over the randomisation process, which would further impact the certainty of the evidence. 

#### Data extraction and management

At least two review authors (TK, LS) independently extracted outcome data from each study using a standardised data collection form. Any discrepancies in the data extracted by the two authors were checked against the original reports, and differences were resolved through discussion and consensus, with recourse to a third author (KW) where necessary. 

We included key characteristics of the studies, including the following information:

- study design, duration of the study, number of study centres and location, study setting and dates of the study;
- information on the participants, including the number randomised, those lost to follow‐up or withdrawn, the number analysed, the age of participants, gender, diagnostic criteria used, inclusion and exclusion criteria for the individual studies;
- details of the intervention, comparator, and concomitant treatments or excluded medications;
- the outcomes specified and reported by the study authors, including the time points;
- funding for the study and any conflicts of interest for the study authors;
- information required to assess the risk of bias in the study, and to enable GRADE assessment of the evidence.

Once the extracted data were checked and any discrepancies had been resolved, a single author (KW) transferred the information to Review Manager 5 (RevMan 2020). 

The primary effect of interest for this review is the effect of treatment assignment (which reflects the outcomes of treatment for people who were assigned to the intervention) rather than a per protocol analysis (the outcomes of treatment only for those who completed the full course of treatment as planned). For the outcomes of interest in this review, we extracted the findings from the studies on an available case basis, i.e. all available data from all participants at each time point, based on the treatment to which they were randomised. This was irrespective of compliance, or whether participants had received the intervention as planned.

In addition to extracting pre‐specified information about study characteristics and aspects of methodology relevant to risk of bias, we extracted the following summary statistics for each trial and outcome:

- For continuous data: the mean values, standard deviation and number of patients for each treatment group at the different time points for outcome measurement. Where change‐from‐baseline data were not available, we extracted the values for endpoint data instead. If values for the individual treatment groups were not reported, where possible we extracted summary statistics (e.g. mean difference) from the studies.
- For binary data: we extracted information on the number of participants experiencing an event, and the number of participants assessed at that time point. If values for the individual treatment groups were not reported, where possible we extracted summary statistics (e.g. risk ratio) from the studies.
- For ordinal scale data: if the data appeared to be normally distributed, or if the analysis performed by the investigators indicated that parametric tests are appropriate, then we treated the outcome measure as continuous data. Alternatively, if data were available, we converted these to binary data for analysis.
- For time‐to‐event data: we did not identify any time‐to‐event data for this review. 

If necessary, we converted data found in the studies to a format appropriate for meta‐analysis, according to the methods described in the *Cochrane Handbook for Systematic Reviews of Interventions* (Handbook 2021). 

We pre‐specified time points of interest for the outcomes in this review. Where studies reported data at multiple time points, we took the longest available follow‐up point within each of the specific time frames. 

#### Assessment of risk of bias in included studies

Two authors (TK, LS) undertook assessment of the risk of bias of the included studies independently. Any discrepancies were resolved by consensus, or through recourse to a third author (KW). The following points were taken into consideration, as guided by the *Cochrane Handbook for Systematic Reviews of Interventions* (Handbook 2011):

- sequence generation;
- allocation concealment;
- blinding;
- incomplete outcome data;
- selective outcome reporting; and
- other sources of bias.

We used the Cochrane risk of bias tool (Handbook 2011), which involves describing each of these domains as reported in the study and then assigning a judgement about the adequacy of each entry: 'low', 'high' or 'unclear' risk of bias. 

#### Measures of treatment effect

We summarised the effects of dichotomous outcomes (e.g. serious adverse effects) as risk ratios (RR) with 95% confidence intervals (CIs). For the key outcomes that we present in the summary of findings tables, we also expressed the results as absolute numbers based on the pooled results and compared to the assumed risk. The assumed baseline risk is the average risk of the control group in the included study (Handbook 2021). For continuous outcomes, we expressed treatment effects as a mean difference (MD) with standard deviation (SD). 

#### Unit of analysis issues

We considered that PPPD could be regarded as a relatively stable condition, therefore cross‐over trials were eligible for inclusion, if the data were reported in a way that allowed for meta‐analysis. If cluster‐randomised trials had been identified then we would have ensured that analysis methods were used to account for clustering in the data (Handbook 2021). However, these study designs were not identified in the review process. We also did not identify any eligible studies that had more than two relevant study groups for comparison. 

#### Dealing with missing data

We planned to contact study authors via email whenever the outcome of interest was not reported, if the methods of the study suggested that the outcome had been measured. However, this was not necessary.

#### Assessment of heterogeneity

We planned to assess clinical heterogeneity by examining the included studies for potential differences between them in the types of participants recruited, interventions or controls used and the outcomes measured. However, as only one study was included, this was not possible. 

#### Assessment of reporting biases

We planned to assess reporting bias as within‐study outcome reporting bias and between‐study publication bias.

##### Outcome reporting bias (within‐study reporting bias)

We planned to assess within‐study reporting bias by comparing the outcomes in the published report against the study protocol or trial registry, whenever this could be obtained. If the protocol or trial registry entry was not available, we compared the outcomes reported to those listed in the methods section. If results are mentioned but not reported adequately in a way that allows analysis (e.g. the report only mentions whether the results were statistically significant or not), bias in a meta‐analysis is likely to occur. If no further information could be found, we noted this as being a 'high' risk of bias when the risk of bias tool was used. If there was insufficient information to judge the risk of bias we noted this as an 'unclear' risk of bias (Handbook 2021). 

##### Publication bias (between‐study reporting bias)

We planned to assess funnel plots if sufficient studies (more than 10) had been available for an outcome. If we had observed asymmetry of the funnel plot, we would have conducted more formal investigation using the methods proposed by Egger 1997. We would also have reported on whether there were any studies identified through trial registries and other sources (Searching other resources), with unpublished reports.

#### Data synthesis

As a single study was included in this review, we were not able to provide a synthesis of results. Please see the protocol for details of how we had planned to synthesise data (Webster 2022b).

#### Subgroup analysis and investigation of heterogeneity

If statistical heterogeneity had been identified for any comparison, we planned to assess this considering the following subgroups:

- different types of intervention, within a specific group;
- use of concomitant treatment.

However, as a single study was included in this review, we were not able to conduct any subgroup analysis. 

#### Sensitivity analysis

We had planned to conduct a small number of sensitivity analyses as part of the review process. However, as a single study was included we were not able to carry these out. More details are available in the protocol for this review (Webster 2022b). 

#### Summary of findings and assessment of the certainty of the evidence

Two independent authors (TK, KW) used the GRADE approach to rate the overall certainty of evidence using GRADEpro GDT (https://gradepro.org/) and the guidance in Chapter 14 of the *Cochrane Handbook for Systematic Reviews of Interventions* (Handbook 2021). Disagreements were resolved through discussion and consensus, or with recourse to a third author if necessary. The certainty of evidence reflects the extent to which we are confident that an estimate of effect is correct, and we applied this in the interpretation of results. There are four possible ratings: high, moderate, low and very low. A rating of high certainty of evidence implies that we are confident in our estimate of effect and that further research is very unlikely to change our confidence in the estimate of effect. A rating of very low certainty implies that any estimate of effect obtained is very uncertain.

The GRADE approach rates evidence from RCTs that do not have serious limitations as high certainty. However, several factors can lead to the downgrading of the evidence to moderate, low or very low. The degree of downgrading is determined by the seriousness of these factors:

- Study limitations (risk of bias)
  - This was assessed using the rating from the Cochrane risk of bias tool for the study or studies included in the analysis. We rated down either one or two levels, depending on the number of domains that had been rated at high or unclear risk of bias. 
- Inconsistency
  - This was assessed using the I^2^ statistic and the P value for heterogeneity for all meta‐analyses, as well as by visual inspection of the forest plot. For results based on a single study we rated this domain as no serious inconsistency.
- Indirectness of evidence
  - We took into account whether there were concerns over the population included in the study or studies for each outcome, as well as whether additional treatments were offered that may impact on the efficacy of the intervention under consideration. 
- Imprecision
  - We took into account the sample size and the width of the confidence interval for each outcome. If the sample size did not meet the optimal information size (i.e. < 400 people for continuous outcomes or < 300 events for dichotomous outcomes), or the confidence interval crossed the small effect threshold we rated down one level. If the sample size did not meet the optimal information size and the confidence interval included both potential harm and potential benefit we rated down twice. We also rated down twice for very tiny studies (e.g. 10 to 15 participants in each arm), regardless of the estimated confidence interval.
- Publication bias
  - We considered whether there were likely to be unpublished studies that may impact on our confidence in the results obtained. 

We used a minimally contextualised approach, and rated the certainty in the interventions having an important effect (Zeng 2021). Where possible, we used agreed minimally important differences (MIDs) for continuous outcomes as the threshold for an important difference. Where no MID was identified, we provide an assumed MID based on agreement between the authors. For dichotomous outcomes, we looked at the absolute effects when rating imprecision, but also took into consideration the GRADE default approach (rating down when a risk ratio crosses 1.25 or 0.80). We have justified all decisions to downgrade the certainty of the evidence using footnotes, and added comments to aid the interpretation of the findings, where necessary. 

We have provided a summary of findings tables for the comparisons:

- talking therapies or stress management versus no treatment;
- vestibular rehabilitation versus no treatment;
- transcranial direct current stimulation versus placebo/no treatment.

We have included all primary outcomes in the summary of findings tables. We planned to prioritise outcomes at the time point three to six months for presentation in the table. We have also included a full GRADE profile for all results (see Table 4).

**1:** Transcranial direct current stimulation versus sham treatment for PPPD

**Certainty assessment**							**№ of participants**		**Effect**		**Certainty**
**№ of studies**	**Study design**	**Risk of bias**	**Inconsistency**	**Indirectness**	**Imprecision**	**Other considerations**	**Transcranial direct current stimulation**	**Sham transcranial direct current stimulation**	**Relative** **(95% CI)**	**Absolute** **(95% CI)**	
**Serious adverse events**											
1	Randomised trial	Serious^a^	Not serious	Not serious^a^	Very serious^b^	None	0/11	0/10	Not estimable	No events occurred in either group.	⨁◯◯◯ Very low
**Disease‐specific health‐related quality of life (assessed with the DHI at 3 months)**											
1	Randomised trial	Serious^a^	Not serious	Not serious^a^	Very serious^c^	None	11	10	—	MD **6 points higher** (5.66 lower to 17.66 higher)	⨁◯◯◯ Very low
**Other adverse events: headache**											
1	Randomised trial	Serious^a^	Not serious	Not serious^a^	Very serious^d^	None	5/11 (45%)	3/10 (30%)	RR 1.52 (0.48 to 4.77)	**156 more per 1000** (from 166 fewer to 700 more)	⨁◯◯◯ Very low
**Other adverse events: sleep disturbance**											
1	Randomised trial	Serious^a^	Not serious	Not serious^a^	Very serious^d^	None	3/11 (27%)	2/10 (20%)	RR 1.36 (0.28 to 6.56)	**72 more per 1000** (from 144 fewer to 800 more)	⨁◯◯◯ Very low
**Other adverse events: dizziness**											
1	Randomised trial	Serious^a^	Not serious	Not serious^a^	Very serious^d^	None	6/11 (55%)	6/10 (60%)	RR 0.91 (0.43 to 1.90)	**54 fewer per 1000** (from 352 fewer to 400 more)	⨁◯◯◯ Very low
**Other adverse events: itchiness**											
1	Randomised trial	Serious^a^	Not serious	Not serious^a^	Very serious^d^	None	6/11 (55%)	4/10 (40%)	RR 1.36 (0.54 to 3.46)	**144 more per 1000** (from 184 fewer to 600 more)	⨁◯◯◯ Very low
**Other adverse events: tingling sensation**											
1	Randomised trial	Serious^a^	Not serious	Not serious^a^	Very serious^d^	None	5/11 (45%)	3/10 (30%)	RR 1.52 (0.48 to 4.77)	**156 more per 1000** (from 166 fewer to 700 more)	⨁◯◯◯ Very low
**Other adverse events: pain at stimulation site**											
1	Randomised trial	Serious^a^	Not serious	Not serious^a^	Very serious^d^	None	3/11 (27%)	3/10 (30%)	RR 0.91 (0.24 to 3.51)	**27 fewer per 1000** (from 228 fewer to 700 more)	⨁◯◯◯ Very low
**Other adverse events: neck pain**											
1	Randomised trial	Serious^a^	Not serious	Not serious^a^	Very serious^d^	None	3/11 (27%)	1/10 (10%)	RR 2.73 (0.34 to 22.16)	**173 more per 1000** (from 66 fewer to 900 more)	⨁◯◯◯ Very low
**Other adverse events: redness of skin**											
1	Randomised trial	Serious^a^	Not serious	Not serious^a^	Very serious^d^	None	3/11 (27%)	1/10 (10%)	RR 2.73 (0.34 to 22.16)	**173 more per 1000** (from 66 fewer to 900 more)	⨁◯◯◯ Very low
**Other adverse events: reduced concentration**											
1	Randomised trial	Serious^a^	Not serious	Not serious^a^	Very serious^d^	None	3/11 (27%)	1/10 (10%)	RR 2.73 (0.34 to 22.16)	**173 more per 1000** (from 66 fewer to 900 more)	⨁◯◯◯ Very low
**Other adverse events: fatigue**											
1	Randomised trial	Serious^a^	Not serious	Not serious^a^	Very serious^d^	None	3/11 (27%)	2/10 (20%)	RR 1.36 (0.28 to 6.56)	**72 more per 1000** (from 144 fewer to 800 more)	⨁◯◯◯ Very low
**Other adverse events: nausea**											
1	Randomised trial	Serious^a^	Not serious	Not serious^a^	Very serious^d^	None	1/11 (9%)	2/10 (20%)	RR 0.45 (0.05 to 4.28)	**110 fewer per 1000** (from 190 fewer to 656 more)	⨁◯◯◯ Very low
**Other adverse events: burning sensation**											
1	Randomised trial	Serious^a^	Not serious	Not serious^a^	Very serious^d^	None	1/11 (9%)	2/10 (20%)	RR 0.45 (0.05 to 4.28)	**110 fewer per 1000** (from 190 fewer to 656 more)	⨁◯◯◯ Very low

DHI: Dizziness Handicap Inventory ^a^This rating includes a partial downgrade for the unclear risk of selection bias (due to concerns over allocation concealment), as well as a partial downgrade for indirectness. This was due to the small sample size not adequately representing the population, and the use of only one specific form of delivery (frequency and duration) of this intervention. ^b^No events occurred in either group and the study was extremely small. We are unable to estimate the relative risk. ^c^Very few participants were included in this study. The confidence interval ranges from the possibility of a trivial effect to a moderate harm from the intervention. ^d^Very few participants were included in this study. The confidence interval ranges from the possibility of a substantial benefit to substantial harm from the intervention.

## Results

### Description of studies

#### Results of the search

The searches in November 2022 retrieved a total of 1803 records. This reduced to 1233 after the removal of duplicates. We screened the titles and abstracts of these 1233 records. We discarded 1174 records and assessed the full text of 59 records. We subsequently excluded 56 records (linked to 43 studies) (see Excluded studies). We identified two ongoing studies, and one study for inclusion in the review. 

A flow chart of study retrieval and selection is provided in Figure 1.

#### Included studies

A single study was included in this review (Im 2022). This randomised controlled trial compared the use of transcranial direct current stimulation (tDCS) to the use of placebo stimulation in 24 people with PPPD. All participants in this study were also offered treatment with a selective serotonin reuptake inhibitor (SSRI) at the start of the study (although two participants took a herbal medicine instead). Participants were diagnosed using the Bárány Society criteria, and had a mean duration of symptoms of around 15 to 18 months. The tDCS was used for 20 minutes at a time, for a total of 15 sessions over a three‐week period, and follow‐up was conducted at three months. The only outcomes of relevance to this review were disease‐specific health‐related quality of life (as measured with the Dizziness Handicap Inventory (DHI)) and adverse effects (as recorded using a patient questionnaire, to be completed after each treatment session). 

We also identified two ongoing trials in this area, which may be relevant for future updates of this review. ChiCTR2000040204 2020 is a study of transcranial magnetic stimulation (compared to sham stimulation) for PPPD. However, the duration of follow‐up for this study is not clear. UMIN000046786 is a study of cognitive behavioural therapy compared to no treatment for chronic dizziness. It is possible that participants in this study will have a variety of causes for their dizziness symptoms. However, one of the outcomes measures appears to relate specifically to PPPD. 

#### Excluded studies

We excluded 43 studies from this review. The main reasons for exclusion are listed below. 

We excluded three studies due to the study design. One was not a randomised controlled trial (UMIN000021150 2016). Two were systematic reviews (Kundakci 2018; Lilios 2021). We checked the reference lists of these articles to ensure that no relevant studies had been missed by our search. 

We excluded 27 studies because the population was incorrect. These studies reported the inclusion of participants with chronic dizziness symptoms, but actually included people with a variety of diagnoses, and not specifically PPPD (Ardiç 2021; Aquaroni 2016; Basta 2011; Basta 2017; Dal 2021; Edelman 2012; Geraghty 2017; ISRCTN52695239 2013; Koganemaru 2017; Kristiansen 2019; Limburg 2021; Manso 2016; Menant 2018; NCT03726112 2018; NCT04425928 2020; NCT04687371 2020; NTR6755 2017; Pavlou 2013; Rinaudo 2021; Rinaudo 2021a; Schlick 2016; Shiozaki 2021; Smaerup 2016; van Vugt 2019; Viziano 2019; Xue 2013; Yardley 2012).

We excluded one study as it assessed an irrelevant intervention (Nada 2019). This study compared vestibular rehabilitation to vestibular rehabilitation plus placebo in a group of people with PPPD. The authors compared the effects of vestibular rehabilitation with a before‐and‐after design, but also assessed whether the addition of a placebo affected outcomes. The intervention of interest in this study was therefore a placebo, rather than an active intervention. 

Five studies used an incorrect comparator ‐ the active intervention was not compared to no intervention or some form of placebo. This included the following:

- ChiCTR2000035073 2020: this ongoing trial compares four different interventions (vestibular rehabilitation, a serotonin‐norepinephrine reuptake inhibitor, transcranial magnetic stimulation and betahistine).
- Herdman 2022: this trial compared an integrated cognitive behavioural therapy (CBT)/vestibular rehabilitation programme to a standard vestibular rehabilitation programme. 
- NCT03029949 2017: this ongoing trial will compare two different kinds of therapy ‐ acceptance and commitment therapy plus vestibular rehabilitation, compared to self‐treatment vestibular rehabilitation using a booklet. 
- Teh 2022: a new, home‐based vestibular rehabilitation programme was compared to an existing hospital‐based rehabilitation programme. 
- Wu 2021: this ongoing trial compares app‐based vestibular rehabilitation to office‐based vestibular rehabilitation.

We excluded seven studies due to insufficient follow‐up. Although these were randomised controlled trials including individuals with PPPD, follow‐up was for less than three months (or 12 weeks). This included the following studies:

- Choi 2021: this study assessed the use of optokinetic stimulation to no treatment (plus concurrent vestibular rehabilitation in both groups), but followed up for only four weeks. 
- Eren 2018: this study assessed the use of non‐invasive vagus nerve stimulation for PPPD (used twice daily, and also at the time of a dizzy episode) compared to no treatment, but follow‐up was only conducted at four weeks. 
- Gordon 2018: this study included people with a variety of vestibular problems, but the majority did have PPPD, and some subgroup data are presented for those with PPPD. However, follow‐up of the intervention (eyeglasses with referential markings) was only assessed at four weeks. 
- NCT05002374 2021: this ongoing study compares the same intervention and comparison as Choi 2021, although it appears to be conducted by a separate author team. The planned follow‐up is also only four weeks. The population will have 'peripheral vestibular dysfunction', therefore may also be inappropriate for the review (but may include some people with PPPD). 
- Shin 2017: this ongoing study will compare a herbal medicine to placebo. However, it is also unclear whether any participants will have PPPD, and the duration of follow‐up is insufficient (eight weeks). 
- Yu 2018: this study included participants with PPPD and compared the use of sertraline to the use of sertraline plus CBT. However, outcomes were only assessed at eight weeks follow‐up. 
- Zhao 2022: this trial compared the use of CBT in addition to standard treatment (medication and vestibular rehabilitation for both groups). However, participants were only followed up until eight weeks. 

### Risk of bias in included studies

See Figure 3 for a summary of the risk of bias for the single included study. 

**3:**
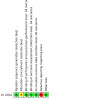
Risk of bias summary (our judgements about each risk of bias item for the included study).

#### Allocation

We rated Im 2022 at unclear risk of selection bias. Although the random sequence was computer‐generated (therefore low‐risk), there was insufficient information regarding measures taken to conceal allocation. We also noted that equal numbers of participants were allocated to the control and intervention groups (without the use of blocked randomisation). We therefore had some concerns that the allocation process may not have been completely random. 

#### Blinding

We rated the study at low risk of both performance and detection bias. Participants and study personnel appeared to be blinded to group allocation, and adequacy of blinding was also confirmed as part of the study ‐ similar numbers of participants in the active treatment and sham treatment groups believed that they were receiving active treatment. The only outcomes of relevance to this review were the DHI and adverse effects ‐ as these were both reported by blinded participants, we considered that the risk of detection bias was low. 

#### Incomplete outcome data

Some participants were missing from the overall analysis. This included one participant in the active group (who did not attend three‐month follow‐up), and two participants in the sham group. Of the participants in the sham group, one did not attend follow‐up at three months and the other was excluded from the analysis due to poor adherence to the (sham) intervention. Therefore, the study has not been analysed with a full 'intention‐to treat' principle. However, this exclusion criterion was pre‐specified, and ‐ as only one participant was affected ‐ we considered it unlikely to have a significant impact on the results. Consequently, we rated this domain at low risk of bias. 

#### Selective reporting

We had some concerns over selective reporting. In the protocol for this study, we noted that the authors planned to include a 'no treatment', healthy control group. The authors confirmed that these participants were also recruited, but were not analysed as part of this article. We have therefore rated this domain at high risk ‐ however, we note that the results included in the review are unlikely to be affected by this issue.  

#### Other potential sources of bias

We did not identify any other concerns with this study. 

### Effects of interventions

See: Table 1; Table 2; Table 3

#### Transcranial direct current stimulation versus sham treatment for persistent postural‐perceptual dizziness (PPPD)

##### Improvement in vestibular symptoms

This outcome was not reported. 

##### Change in vestibular symptoms

This outcome was not reported. 

##### Serious adverse events

Adverse events were reported by participants using a questionnaire, which was to be completed after each session of transcranial direct current stimulation. We contacted the study authors, who confirmed that no serious adverse events occurred in either group during the course of the study (0/11 in the intervention group, 0/10 in the sham treatment group; relative effect not estimable; 1 study; 21 participants; very low‐certainty evidence). 

##### Disease‐specific health‐related quality of life

This outcome was assessed using the Dizziness Handicap Inventory (DHI). Scores on this scale range from 1 to 100, with higher scores representing worse quality of life. 

###### 3 to < 6 months

At three months, DHI scores were slightly higher (worse) in people who had received transcranial direct current stimulation, as compared to those who received sham treatment (mean difference 6 points, 95% CI ‐5.66 to 17.66; 1 study; 22 participants; very low‐certainty evidence; Analysis 1.1). The minimally important difference on the DHI has been suggested to be between 11 and 18 points (Jacobsen 1990; Tamber 2009). 

###### 6 to ≤ 12 months and > 12 months

This outcome was not reported at these time periods. 

##### Generic health‐related quality of life

This outcome was not reported. 

##### Other adverse effects

We pre‐specified a number of specific adverse effects of interest in this review. However, at the time of writing the protocol we had not anticipated identifying evidence on transcranial direct current stimulation, and the specified adverse effects may not be relevant to this intervention. We have therefore included an assessment of all adverse effects reported by the authors of this study. Due to the small number of people included in this study, and the relatively infrequent occurrence of adverse effects, the confidence intervals are extremely wide, and we cannot draw any conclusions from these analyses. However, for completeness we have displayed a comparison of the individual listed adverse effects for each group (see Analysis 1.2). 

## Discussion

### Summary of main results

We did not identify any studies that met our original inclusion criteria for this review ‐ comparing talking therapies or vestibular rehabilitation to no treatment in people with persistent postural‐perceptual dizziness (PPPD), and conducting follow‐up at three months or later. However, we did identify an unanticipated intervention (transcranial direct current stimulation), which otherwise met the criteria for inclusion in this review and would be considered as a non‐pharmacological intervention. We therefore decided to include the results of this study in the review, to present as complete a picture as possible of the current evidence base for non‐pharmacological interventions for PPPD. 

The included study compared the use of transcranial direct current stimulation (administered for 20 minutes at a time, on 15 occasions over a three‐week period), to sham treatment in 24 people with PPPD. Data were reported for only a few of our review outcomes. Transcranial direct current stimulation may result in little or no difference in disease‐specific health‐related quality of life (as measured with the Dizziness Handicap Inventory (DHI)) at three months, but the evidence is very uncertain. Adverse events were assessed as part of this study. No participants in either group reported serious adverse events. The evidence is very uncertain about the occurrence of more minor adverse effects. 

### Overall completeness and applicability of evidence

We identified only one study that was suitable for inclusion in this review, and this considered an intervention that ‐ to our knowledge ‐ is not in widespread use for PPPD (transcranial direct current stimulation). This study only considered some of our outcomes of interest, so the efficacy of this intervention for vestibular symptoms and generic quality of life is unknown. Data on adverse effects were also sparse, due to the small number of participants in this study. Furthermore, follow‐up was only conducted at three months, so we do not know if there are any longer‐term effects of this intervention. It should be noted that participants in both arms of this study also started treatment with a selective serotonin reuptake inhibitor (SSRI) at the time of enrolling in the trial. This may have affected the results, as any beneficial (or harmful) effects from medical treatment may mask any additional effects of the transcranial direct current stimulation.

Although we did identify a number of randomised controlled trials (RCTs) that aimed to assess the efficacy of interventions for PPPD, these were not suitable for inclusion in this review due to two major concerns. Firstly, a number of studies compared two different interventions, without the use of an appropriate placebo or 'no treatment' control group. For a relatively new diagnostic category, this is surprising. To establish a 'gold standard' treatment for PPPD, we must first be certain that any effects seen are better than no treatment, and this requires assessment in a randomised trial. 

A second issue seen with the studies that we did identify is the relatively short duration of follow‐up. A small number of RCTs were found that compared an active intervention to no treatment for PPPD, but the follow‐up time was only four to eight weeks. For a chronic condition, we considered this duration of follow‐up to be insufficient. A short‐term change in symptoms may not accurately reflect the efficacy of these treatments over a longer time period, and therefore is unlikely to be helpful when weighing up different treatment options. 

### Quality of the evidence

We assessed the certainty of the evidence using the GRADE approach. Overall, we rated the certainty of the evidence as very low for all outcomes. This was due to the small numbers of participants in the trial (only 24 people), leading to wide confidence intervals, and imprecision in the overall effect estimates. 

### Potential biases in the review process

As described above, we did exclude some studies from this review as they did not meet our inclusion criteria. The omission of studies with short follow‐up, or of those studies that did not include an appropriate placebo or 'no treatment' arm, could be considered by some as a source of bias in this review, although it is in accordance with our protocol (Webster 2022b). 

In keeping with our protocol, we only included studies where participants had received a diagnosis of PPPD using the Bárány society criteria. This may have led to the omission of studies that predated these criteria, and recruited participants with different ‐ but related ‐ diagnoses, such as chronic subjective dizziness or phobic postural vertigo. This could be regarded as a bias in the review process. Nonetheless, we considered it vital to focus the review on those with a definitive diagnosis of PPPD, to assess the current evidence for this specific condition.

### Agreements and disagreements with other studies or reviews

There are few published reviews that consider the efficacy and harms of interventions for PPPD. We did not identify any other systematic reviews that have addressed this question. However, we note the findings of a recent narrative review on this topic, which also highlights the lack of RCTs in this field (Staab 2020). Results from a single RCT were included (Yu 2018), which considered a non‐pharmacological intervention (cognitive behavioural therapy) as an adjunct to sertraline treatment (an SSRI) for PPPD. This study was excluded from our review due to an insufficient follow‐up duration (only eight weeks). 

## Authors' conclusions

**Implications for practice**

There is very limited evidence regarding the use of non‐pharmacological interventions for persistent postural‐perceptual dizziness (PPPD). Many of the studies in this area have not compared interventions with an appropriate control group, or have conducted very short follow‐up. People with PPPD and healthcare professionals working in this area should be aware of this uncertainty when selecting treatments for this condition. 

**Implications for research**

Further work is needed in this area to identify whether any interventions are effective in the treatment of PPPD, and whether they are associated with any harms. The following conclusions relate to the evidence identified in this review and a companion review on pharmacological interventions for PPPD (Webster 2022a):

- Authors of future studies should ensure that the agreed diagnostic criteria for PPPD are used to identify study participants (Appendix 1). 
- There is currently no evidence from randomised controlled trials to support a 'gold standard' treatment for PPPD, although some interventions are in widespread use. Placebo‐controlled trials are therefore vital to identify the potential efficacy of interventions for PPPD. Comparison with other interventions (of unknown efficacy) does not allow firm conclusions to be drawn. 
- PPPD is a chronic condition, with symptoms that can recur over months or years. This needs to be considered when designing clinical trials in this area, and determining the appropriate duration of follow‐up. A number of studies were found in the literature that conducted short‐term follow‐up and assessed outcomes at four or eight weeks. We considered that it is not possible to draw conclusions about the efficacy of these interventions with such short follow‐up. We would advocate that authors of future studies plan for at least six months of follow‐up to establish whether interventions have benefit for the long‐term symptoms of this disorder. 
- As with other balance disorders, there should be agreement about which outcomes to measure in studies of PPPD, and how to measure them. There should also be agreement about what size of a difference in symptoms would be meaningful and important to people with this condition. This can only be achieved through collaboration between people with PPPD, healthcare professionals and researchers, and development of a core outcome set would be advantageous. 

## History

Protocol first published: Issue 3, 2022

## Appendices

### Appendix 1. Diagnostic criteria for PPPD

The Bárány Society criteria for PPPD (Staab 2017):

1. One or more symptoms of dizziness, unsteadiness or non‐spinning vertigo, present on most days for three months or more:
  - symptoms are persistent, but wax and wane;
  - symptoms tend to increase as the day progresses, but may not be active throughout the entire day;
  - momentary flares may occur spontaneously or with sudden movements.
2. Symptoms are present without specific provocation, but are exacerbated by:
  - upright posture;
  - active or passive motion without regard to direction or position; and
  - exposure to moving visual stimuli or complex visual patterns, although these three features may not be equally provocative.
3. The disorder usually begins shortly after an event that causes acute vestibular symptoms or problems with balance, though less commonly it develops slowly. Precipitating events include acute, episodic or chronic vestibular syndromes, other neurologic or medical illness, and psychological distress:
  - when triggered by an acute or episodic precipitant, symptoms typically settle into the pattern of criterion 1 as the precipitant resolves, but may occur intermittently at first, and then consolidate into a persistent course;
  - when triggered by a chronic precipitant, symptoms may develop slowly and worsen gradually.
4. Symptoms cause significant distress or functional impairment.
5. Symptoms are not better attributed to another disease or disorder.

### Appendix 2. Trustworthiness Screening Tool

This screening tool has been developed by Cochrane Pregnancy and Childbirth. It includes a set of predefined criteria to select studies that, based on available information, are deemed to be sufficiently trustworthy to be included in the analysis. These criteria are:

**Research governance**

- Are there any retraction notices or expressions of concern listed on the Retraction Watch Database relating to this study?
- Was the study prospectively registered (for those studies published after 2010)? If not, was there a plausible reason?
- When requested, did the trial authors provide/share the protocol and/or ethics approval letter?
- Did the trial authors engage in communication with the Cochrane Review authors within the agreed timelines?
- Did the trial authors provide IPD data upon request? If not, was there a plausible reason?

**Baseline characteristics**

- Is the study free from characteristics of the study participants that appear too similar (e.g. distribution of the mean (SD) excessively narrow or excessively wide, as noted by Carlisle 2017)?

**Feasibility**

- Is the study free from characteristics that could be implausible? (e.g. large numbers of women with a rare condition (such as severe cholestasis in pregnancy) recruited within 12 months);
- In cases with (close to) zero losses to follow‐up, is there a plausible explanation?

**Results**

- Is the study free from results that could be implausible? (e.g. massive risk reduction for main outcomes with small sample size)?
- Do the numbers randomised to each group suggest that adequate randomisation methods were used (e.g. is the study free from issues such as unexpectedly even numbers of women ‘randomised’ including a mismatch between the numbers and the methods, if the authors say ‘no blocking was used’ but still end up with equal numbers, or if the authors say they used ‘blocks of 4’ but the final numbers differ by 6)?

Studies assessed as being potentially ‘high risk’ will be not be included in the review. Where a study is classified as ‘high risk’ for one or more of the above criteria we will attempt to contact the study authors to address any possible lack of information/concerns. If adequate information remains unavailable, the study will remain in ‘awaiting classification’ and the reasons and communications with the author (or lack of) described in detail.

The process is described in full in Figure 2.

### Appendix 3. Search strategies

**Table:**

**CENTRAL (CRS)**	**MEDLINE (Ovid)**	**Embase (Ovid)**
1 MESH DESCRIPTOR Dizziness EXPLODE ALL AND CENTRAL:TARGET 809 2 MESH DESCRIPTOR Vertigo AND CENTRAL:TARGET 509 3 MESH DESCRIPTOR Postural Balance AND CENTRAL:TARGET 0 4 MESH DESCRIPTOR Vestibular Diseases AND CENTRAL:TARGET 0 5 MESH DESCRIPTOR Motion perception EXPLODE ALL AND CENTRAL:TARGET 299 6 #1 OR #2 OR #3 OR #4 OR #5 1515 7 MESH DESCRIPTOR Chronic Disease EXPLODE ALL AND CENTRAL:TARGET 13660 8 (chronic or persistent or "long term" or longstanding):AB,TI,TO AND CENTRAL:TARGET 245806 9 #7 OR #8 247437 10 #6 AND #9 190 11 (chronic or persistent OR persisting or "long term" or longstanding) adj6 (dizziness OR dizzy OR vertigo OR vestibular):AB,TI,TO AND CENTRAL:TARGET 294 12 #10 OR #11 405 13 >2009:YR AND CENTRAL:TARGET 1020392 14 #12 AND #13 269 15 MESH DESCRIPTOR Perceptual Disorders EXPLODE ALL AND INSEGMENT 175 16 #6 AND #15 3 17 (persistent adj3 (perceptual or postural) adj3 (dizziness or vertigo)):AB,EH,KW,KY,MC,MH,TI,TO AND CENTRAL:TARGET 12 18 (PPPD AND (dizziness or vertigo or vestibular)):AB,EH,KW,KY,MC,MH,TI,TO AND CENTRAL:TARGET 11 19 #14 OR #16 OR #17 OR #18 273	MEDLINE (Ovid MEDLINE® Epub Ahead of Print, In‐Process & Other Non‐Indexed Citations, Ovid MEDLINE® Daily and Ovid MEDLINE®) 1946 to present 1 exp Dizziness/ 5763 2 Vertigo/ 10478 3 Postural Balance/ 25602 4 Vestibular Diseases/ 4511 5 exp Motion perception/ 16966 6 1 or 2 or 3 or 4 or 5 59217 7 exp Chronic Disease/ 271574 8 6 and 7 847 9 ((persistent or chronic or persisting or "long term" or longstanding) adj6 (dizziness or dizzy or vertigo or vestibular)).ab,ti. 1765 10 8 or 9 2439 11 (201* or 202*).yr. 13228363 12 10 and 11 1383 13 Perceptual Disorders/ 6739 14 13 and 10 11 15 (persistent adj3 (perceptual or postural) adj3 (dizziness or vertigo)).ab,ti. 102 16 (PPPD and (dizziness or vertigo or vestibular)).ab,ti. 75 17 12 or 14 or 15 or 16 1389 18 randomized controlled trial.pt. 545668 19 controlled clinical trial.pt. 94445 20 randomized.ab. 536242 21 placebo.ab. 222027 22 drug therapy.fs. 2382579 23 randomly.ab. 367234 24 trial.ab. 570881 25 groups.ab. 2255489 26 18 or 19 or 20 or 21 or 22 or 23 or 24 or 25 5137324 27 exp animals/ not humans.sh. 4894687 28 26 not 27 4469024 29 17 and 28 494	Embase 1974 to present 1 exp dizziness/ 84247 2 vertigo/ 47669 3 body equilibrium/ 20518 4 vestibular disorder/ 9404 5 exp movement perception/ 13785 6 1 or 2 or 3 or 4 or 5 164913 7 exp chronic disease/ 190351 8 6 and 7 1397 9 ((persistent or chronic or persisting or longstanding or "long term") adj6 (dizziness or dizzy or vertigo or vestibular)).ab,ti. 2406 10 8 or 9 3628 11 (201* or 202*).yr. 17776219 12 10 and 11 2234 13 exp perception disorder/ 35625 14 10 and 13 45 15 (persistent adj3 (perceptual or postural) adj3 (dizziness or vertigo)).ab,ti. 132 16 (PPPD and (dizziness or vertigo or vestibular)).ab,ti. 101 17 12 or 14 or 15 or 16 2248 18 Randomized controlled trial/ 678551 19 Controlled clinical study/ 464183 20 Random$.ti,ab. 1711730 21 randomization/ 91931 22 intermethod comparison/ 275752 23 placebo.ti,ab. 330279 24 (compare or compared or comparison).ti. 547713 25 ((evaluated or evaluate or evaluating or assessed or assess) and (compare or compared or comparing or comparison)).ab. 2377142 26 (open adj label).ti,ab. 91422 27 ((double or single or doubly or singly) adj (blind or blinded or blindly)).ti,ab. 249007 28 double blind procedure/ 188384 29 parallel group$1.ti,ab. 28187 30 (crossover or cross over).ti,ab. 112909 31 ((assign$ or match or matched or allocation) adj5 (alternate or group$1 or intervention$1 or patient$1 or subject$1 or participant$1)).ti,ab. 364054 32 (assigned or allocated).ti,ab. 429177 33 (controlled adj7 (study or design or trial)).ti,ab. 389388 34 (volunteer or volunteers).ti,ab. 261134 35 human experiment/ 555942 36 trial.ti. 340283 37 18 or 19 or 20 or 21 or 22 or 23 or 24 or 25 or 26 or 27 or 28 or 29 or 30 or 31 or 32 or 33 or 34 or 35 or 36 5534662 38 (random$ adj sampl$ adj7 ("cross section$" or questionnaire$1 or survey$ or database$1)).ti,ab. 12284 39 comparative study/ or controlled study/ 9044969 40 randomi?ed controlled.ti,ab. 326332 41 randomly assigned.ti,ab. 144742 42 39 or 40 or 41 9230030 43 38 not 42 8726 44 Cross‐sectional study/ 437766 45 randomized controlled trial/ or controlled clinical study/ or controlled study/ 8500635 46 (randomi?ed controlled or control group$1).ti,ab. 993836 47 45 or 46 8866090 48 44 not 47 284171 49 (((case adj control$) and random$) not randomi?ed controlled).ti,ab. 18978 50 (Systematic review not (trial or study)).ti. 187767 51 (nonrandom$ not random$).ti,ab. 17282 52 "Random field$".ti,ab. 2590 53 (random cluster adj3 sampl$).ti,ab. 1386 54 (review.ab. and review.pt.) not trial.ti. 929038 55 "we searched".ab. 62552 56 review.ti. or review.pt. 3133557 57 55 and 56 38528 58 "update review".ab. 119 59 (databases adj4 searched).ab. 45639 60 (rat or rats or mouse or mice or swine or porcine or murine or sheep or lambs or pigs or piglets or rabbit or rabbits or cat or cats or dog or dogs or cattle or bovine or monkey or monkeys or trout or marmoset$1).ti. and animal experiment/ 1123962 61 43 or 48 or 49 or 50 or 51 or 52 or 53 or 54 or 57 or 58 or 59 1388815 62 37 not 61 5249339 63 17 and 62 551

## Data and analyses

**Comparison 1:** Transcranial direct current stimulation versus placebo

Outcome or subgroup title	No. of studies	No. of participants	Statistical method	Effect size
1.1 Disease‐specific health‐related quality of life	1	22	Mean Difference (IV, Random, 95% CI)	6.00 [‐5.66, 17.66]
1.2 Other adverse events	1		Risk Ratio (M‐H, Random, 95% CI)	Subtotals only
1.2.1 Headache	1	21	Risk Ratio (M‐H, Random, 95% CI)	1.52 [0.48, 4.77]
1.2.2 Sleep disturbance	1	21	Risk Ratio (M‐H, Random, 95% CI)	1.36 [0.28, 6.56]
1.2.3 Dizziness	1	21	Risk Ratio (M‐H, Random, 95% CI)	0.91 [0.43, 1.90]
1.2.4 Itchiness	1	21	Risk Ratio (M‐H, Random, 95% CI)	1.36 [0.54, 3.46]
1.2.5 Tingling sensation	1	21	Risk Ratio (M‐H, Random, 95% CI)	1.52 [0.48, 4.77]
1.2.6 Pain at stimulation site	1	21	Risk Ratio (M‐H, Random, 95% CI)	0.91 [0.24, 3.51]
1.2.7 Neck pain	1	21	Risk Ratio (M‐H, Random, 95% CI)	2.73 [0.34, 22.16]
1.2.8 Redness of skin	1	21	Risk Ratio (M‐H, Random, 95% CI)	2.73 [0.34, 22.16]
1.2.9 Reduced concentration	1	21	Risk Ratio (M‐H, Random, 95% CI)	2.73 [0.34, 22.16]
1.2.10 Fatigue	1	21	Risk Ratio (M‐H, Random, 95% CI)	1.36 [0.28, 6.56]
1.2.11 Nausea	1	21	Risk Ratio (M‐H, Random, 95% CI)	0.45 [0.05, 4.28]
1.2.12 Burning sensation	1	21	Risk Ratio (M‐H, Random, 95% CI)	0.45 [0.05, 4.28]

## Characteristics of studies

### Characteristics of included studies [ordered by study ID]

**Table:** Im 2022

***Study characteristics***		
Methods	Randomised, double‐blind, 2‐arm, parallel‐group, controlled trial with 3 weeks duration of treatment and follow‐up to a maximum of 3 months	
Participants	**Setting:** Participants were recruited from a neurology outpatient clinic of a single centre in South Korea **Sample size:** **Number randomised:** 24 participants **Number completed:** 21 participants (completed 3‐month follow‐up) **Participant baseline characteristics:** Baseline characteristics for the control group are only reported for those who were analysed (11 out or 12 participants) **Age:** Intervention group: 47.8 years (SD 13.0) Control group: 51.7 years (SD 13.1) **Gender:** Intervention group: 8 females (67%): 4 males (33%) Control group: 7 females (64%): 4 males (34%) **Duration of disease:** Intervention group: 17.6 months (SD 396.3 days)* Control group: 14.8 months (SD 348 days)* **DHI score at baseline:** Intervention group: 34.3 points (SD 15.9) Control group: 35.3 points (SD 14.2) *The authors have confirmed that the SD was reported in days, rather than months **Inclusion criteria:** Men and women between the ages of 18 and 69 who met the diagnostic criteria for persistent postural‐perceptual dizziness (PPPD) **Exclusion criteria:** Neurological disorders other than PPPD, psychiatric disorders (e.g. generalised anxiety disorder or major depressive disorder), contraindications to transcranial direct current stimulation (e.g. metallic implants in the head or history of seizure), history of head trauma with loss of consciousness, or pregnancy. People who had previous used transcranial direct current stimulation were also excluded. People who were non‐adherent to the treatment (< 12 out of 15 sessions) were excluded from the analysis. **Diagnostic criteria used for PPPD:** Diagnostic criteria of PPPD, established by the Classification Committee of the Bárány Society. A single neurologist made the diagnosis for all participants.	
Interventions	**Intervention (n = 12 randomised, n = 11 completed 3‐month follow‐up)** Transcranial direct current stimulation was carried out using the YDS‐301 N device. The anodal electrode was placed over the left dorsolateral prefrontal cortex and the cathodal electrode was placed over the right dorsolateral prefrontal cortex. The current was increased to 2.0 mA (a current density of 0.07 mA/cm^2^) over a period of 30 seconds, maintained at 2.0 mA for 19 minutes, then reduced to 0 mA over 30 seconds. A total of 15 sessions were to be carried out over a 3‐week period, with a maximum of 1 session per day. **Control (n = 12 randomised, n = 10 completed 3‐month follow‐up)** Sham stimulation was carried out. The device was positioned in the same way as for the active intervention group, but the current was increased to 2.0 mA over 30 seconds, then reduced again over the next 30 seconds. **Background interventions used for all participants:** Participants were recommended to start and continue maintenance treatment with SSRIs for at least 3 weeks prior to and during the study period. The majority of participants started taking an SSRI (20 participants with escitalopram (8.82 ± 4.52 mg per day) and 1 with sertraline (25 mg per day). Two participants took a herbal medicine instead (hypericum extracts known for antidepressant effects).	
Outcomes	**Primary outcomes relevant to this review:** **Improvement in vestibular symptoms** Not reported **Change in vestibular symptoms** Not reported **Serious adverse events** A number of side effects are stated in the article, and the authors state that "Participants were asked to fill out a survey on side effects after each session". **Secondary outcomes relevant to this review:** **Disease‐specific health‐related quality of life** Assessed using the Dizziness Handicap Inventory at 3‐month follow‐up **Generic health‐related quality of life** Not reported **Other adverse effects** A number of potential adverse effects are listed in the article **Other outcomes reported in the study:** Activities‐specific Balance Confidence (ABC) scale Hamilton Depression Rating Scale (HDRS) Hamilton Anxiety Rating Scale (HARS) Brain SPECT Imaging	
Notes	**Research integrity checklist:** No retractions or expressions of concern were identified The trial was registered with cris.nih.go.kr, identifier: KCT0005068 Baseline characteristics were not excessively similar Plausible dropout is reported No implausible results were noted The numbers allocated to each group were identical, and there was no report of blocked randomisation	
***Risk of bias***		
**Bias**	**Authors' judgement**	**Support for judgement**
Random sequence generation (selection bias)	Low risk	Quote: "Randomization was performed using a computer‐based random number‐producing algorithm by a research staff who was not involved in the data collection (HJ)." Comment: computerised random sequence generation.
Allocation concealment (selection bias)	Unclear risk	Comment: the authors confirmed that randomisation and allocation to groups was carried out by one individual, who was not involved in the collection of outcome data or analysis of the trial results. It is not clear, however, what steps were taken to ensure that implementation of the random sequence was carried out accurately and without interference.
Blinding of participants and personnel (performance bias) All outcomes	Low risk	Quote: "Adequacy of blinding to treatment condition was assessed at the immediate follow‐up by asking the participants to guess the treatment condition (active, sham, or do not know) that they were assigned to. In the active group, 6 participants (50%) answered that they received active treatment (correctly guessing their treatment group) and 6 participants (50%) answered, 'don't know'. In the sham group, 7 participants (63.63%) answered that they received active treatment (incorrectly guessing their treatment group), 2 participants (18.18%) answered that they received sham treatment (correctly guessing their treatment group), and 2 participants (18.18%) answered, 'don't know'." Comment: participants were blinded to their allocated intervention, and it appears that the methods used for blinding were adequate.
Blinding of outcome assessment (detection bias) All outcomes	Low risk	Comment: outcomes included in this review were reported by (blinded) participants, therefore should not be subject to detection bias.
Incomplete outcome data (attrition bias) All outcomes	Low risk	Quote: "Among 24 participants who were randomized into active (n=12) or sham (n=12) groups, 23 participants were included in the final analysis after excluding one participant in the sham group due to low adherence to tDCS [transcranial direct current stimulation] treatment" Comment: all participants completed immediate follow‐up but 2 did not complete 1‐ and 3‐month follow‐up visits (1 in the active treatment group, 1 in the comparator group). Additionally, in the comparator condition a further person was excluded from the analysis due to low adherence to treatment. However, overall the extent of dropout was relatively low, and unlikely to have a large impact on the estimated effect sizes.
Selective reporting (reporting bias)	High risk	Comment: we note some deviation from the study protocol, as a no treatment control group was also planned to be recruited. This is not explained in the article. We understand from the authors that the control group were recruited, but the data were not analysed. However, this is unlikely to impact on the results included in this review.
Other bias	Low risk	Comment: no other concerns were identified.

DHI: Dizziness Handicap Inventory PPPD: persistent postural‐perceptual dizziness SD: standard deviation SPECT: single‐photon emission computerised tomography SSRI: selective serotonin reuptake inhibitor tDCS: transcranial direct current stimulation

### Characteristics of excluded studies [ordered by study ID]

**Table:**

Study	Reason for exclusion
Aquaroni 2016	This RCT included older adults with vestibular disorders, but not specifically participants with PPPD.
Ardiç 2021	The population was incorrect. Participants had chronic unilateral vestibular dysfunction and persistent dizziness, but did not have a diagnosis of PPPD.
Basta 2011	The population included in this RCT had causes of vestibular dysfunction other than PPPD.
Basta 2017	The population included in this RCT did not have PPPD; they were diagnosed with other vestibular disorders.
ChiCTR2000035073 2020	The comparator for this 4‐arm RCT is incorrect. Participants are allocated to either 1) cerebellar transcranial magnetic stimulation, 2) SNRI medication, 3) vestibular rehabilitation or 4) oral betahistine. No control group/placebo group was included.
Choi 2021	This is an RCT including people with PPPD. However, the duration of follow‐up was insufficient (4 weeks). The intervention assessed was optokinetic stimulation.
Dal 2021	The population was incorrect. Participants were diagnosed with a unilateral vestibular disorder, not PPPD.
Edelman 2012	The population was incorrect. Participants were diagnosed with chronic subjective dizziness, not PPPD. In addition, outcomes were only assessed at 4‐week follow‐up.
Eren 2018	This was an RCT of non‐invasive vagus nerve stimulation for people with PPPD. However, the duration of follow‐up was only 4 weeks.
Geraghty 2017	The population was incorrect. This RCT included people with dizziness symptoms, but not specifically PPPD.
Gordon 2018	This study included participants with symptoms of chronic dizziness, and assessed the use of eyeglasses with referential marks on the lenses. The majority of these people did have PPPD, and some outcome data are reported specifically for this subgroup. However, follow‐up was only conducted at 4 weeks.
Herdman 2022	This RCT was excluded due to the wrong comparator. The intervention (cognitive behavioural therapy informed vestibular rehabilitation) was compared to standard vestibular rehabilitation.
ISRCTN52695239 2013	The population is incorrect. This ongoing study will include participants with a number of diagnoses (including vertigo and dizziness of unknown cause, Ménière's disease and psychogenic dizziness). It will not specifically include those with PPPD.
Koganemaru 2017	The population was incorrect. Participants had unilateral vestibular dysfunction due to conditions including vestibular neuritis, Ramsay Hunt syndrome and acoustic neuroma. Participants did not have PPPD.
Kristiansen 2019	The population is incorrect. Participants in this ongoing trial must have an acute onset of dizziness, which has lasted at least 3 months, but not specifically PPPD.
Kundakci 2018	This is a systematic review. The reference list has been checked to ensure that any relevant studies were identified.
Lilios 2021	This is a systematic review. The reference list has been checked to ensure that any relevant studies were identified.
Limburg 2021	The population is not appropriate for this review. Participants had chronic dizziness resulting from a vestibular disorder, but did not have a diagnosis of PPPD. The comparator was also not correct, as this study compared two different types of vestibular rehabilitation.
Manso 2016	The population was incorrect. Participants had chronic dizziness, with at least one episode of dizziness every month. They did not have a diagnosis of PPPD.
Menant 2018	The population was incorrect. This trial recruited older adults who had experienced at least one significant episode of dizziness over the previous 12 months, not people with PPPD.
Nada 2019	This was a randomised trial of adults with a diagnosis of PPPD. However, the intervention was incorrect: this trial randomised participants to receive vestibular rehabilitation alone, or vestibular rehabilitation plus a placebo tablet. The authors assessed whether the addition of a placebo affected outcomes, and also made a before‐and‐after comparison of both groups following vestibular rehabilitation.
NCT03029949 2017	This is an ongoing study that will include participants with PPPD. However, the comparator is incorrect ‐ the study will compare group acceptance and commitment therapy with vestibular rehabilitation, to self‐treatment vestibular rehabilitation (using a self‐guide booklet).
NCT03726112 2018	This study has been temporarily suspended. However, the population is incorrect (it will recruit participants with chronic dizziness, but not necessarily PPPD), and follow‐up will only be for 8 weeks.
NCT04425928 2020	The population is incorrect: this study included people with chronic, unilateral peripheral vestibular disorders, not PPPD. Follow‐up was also only 4 weeks.
NCT04687371 2020	The population is incorrect: participants in this study had unilateral peripheral vestibular hypofunction.
NCT05002374 2021	This is an ongoing trial. The population is not completely clear, but may include at least some individuals with PPPD. The study will compare vestibular rehabilitation alone to vestibular rehabilitation with optokinetic stimulation. However, the duration of follow‐up is insufficient (4 weeks).
NTR6755 2017	The population is not appropriate. This study will include participants with medically unexplained symptoms, not PPPD.
Pavlou 2013	The population was not appropriate. This study recruited participants with peripheral vestibular disorders (predominantly vestibular neuritis or vestibular migraine), not PPPD.
Rinaudo 2021	The population is incorrect. Participants had stable and isolated peripheral vestibular hypofunction, not a diagnosis of PPPD.
Rinaudo 2021a	The population is incorrect. Participants had vestibular hypofunction (mainly vestibular neuritis or labyrinthitis), not PPPD.
Schlick 2016	The population is incorrect. Participants had chronic dizziness, but not necessarily PPPD. The comparator was also incorrect, as this study compares two different types of vestibular rehabilitation.
Shin 2017	This study will recruit participants with chronic dizziness, including Ménière's disease, psychogenic dizziness or dizziness of unknown cause. This may include some participants with PPPD. However, the intervention is not appropriate for this review (a Chinese herbal medicine), and follow‐up is insufficient (8 weeks).
Shiozaki 2021	The population is incorrect. Participants had unilateral or bilateral peripheral vestibular hypofunction with symptoms of dizziness and imbalance for at least 6 months, but not PPPD.
Smaerup 2016	The population is not appropriate. This study recruited people aged over 65 years with peripheral, central or mixed vestibular dysfunction, but not specifically PPPD.
Teh 2022	The comparator for this RCT is incorrect. A new, home‐based vestibular rehabilitation programme was compared to a hospital‐based rehabilitation programme.
UMIN000021150 2016	This study is not an RCT.
van Vugt 2019	This RCT recruited older adults with a chronic vestibular syndrome, but not specifically PPPD.
Viziano 2019	The population is incorrect. This study recruited participants with chronic vestibular hypofunction, not specifically PPPD.
Wu 2021	The comparator for this ongoing trial is not relevant. The intervention (WeChat, app‐based, vestibular rehabilitation) will be compared to standard, in‐person vestibular rehabilitation.
Xue 2013	This study did not specifically recruit individuals with PPPD. The intervention was also not specified in our review protocol, and follow‐up is insufficient (maximum of 8 weeks).
Yardley 2012	The population is not appropriate. This study recruited participants with chronic dizziness, but not specifically PPPD.
Yu 2018	The duration of follow‐up is insufficient. This study compared the use of sertraline plus CBT to sertraline alone in participants with PPPD.
Zhao 2022	This trial compared the use of CBT in addition to standard treatment (medication and vestibular rehabilitation for both groups). However, the duration of follow‐up for this trial was insufficient as participants were only followed up until 8 weeks.

CBT: cognitive behavioural therapy PPPD: persistent postural‐perceptual dizziness RCT: randomised controlled trial

### Characteristics of ongoing studies [ordered by study ID]

**Table:** ChiCTR2000040204 2020

Study name	'Clinical effect of cerebellar repetitive transcranial magnetic stimulation versus the right prefrontal in treatment of persistent postural‐perceptual dizziness: a randomised controlled trial'
Methods	Parallel‐group randomised controlled trial
Participants	**Inclusion criteria:** People with PPPD, according to the Bárány Society criteria, aged 18 to 80 years old **Exclusion criteria:** Other causes of vestibular dysfunction, including Ménière's disease, vestibular migraine, benign paroxysmal positional vertigo or other acute vestibular syndromes. Central nervous system causes of dizziness. Anxiety, depression or other serious mental disorders. Pregnancy or lactation. Contraindications to transcranial magnetic stimulation.
Interventions	Two groups will receive the active intervention. A coil for transcranial magnetic stimulation will be placed either in the posterior fossa, under the occipital region (for cerebellar stimulation) or in the dorsolateral area of the right prefrontal lobe (for prefrontal lobe stimulation). One thousand pulses will be continuously stimulated at low frequency (1 Hz), 10 times per day. The control group will receive sham treatment, but this is not further described.
Outcomes	Dizziness Handicap Inventory (DHI) Hamilton Anxiety Scale Hamilton Depression Scale Pittsburgh Sleep Quality Index Clinical Global Impression ‐ Severity score Clinical Global Impression ‐ Improvement score These measures will be performed before and after treatment, but the timing is not specified
Starting date	Planned study dates: November 2020 to December 2021
Contact information	Xidan Li lixidandan@sina.com
Notes	We are uncertain if the follow‐up for this study will be sufficient for inclusion in future updates of this review

**Table:** UMIN000046786

Study name	'Verification of the effect of cognitive behavioral therapy randomized controlled trials for patients with chronic dizziness'
Methods	Parallel‐group randomised controlled trial
Participants	**Inclusion criteria:** People with dizziness for at least 3 months, aged 20 to 65 years old **Exclusion criteria:** Dementia or cognitive decline. Severe depression, bipolar disorder, schizophrenia Estimated sample size: 60 participants
Interventions	The intervention group will receive cognitive behavioural therapy (no further details provided) The control group will receive standard care
Outcomes	Dizziness Handicap Inventory Niigata PPPD Questionnaire Hospital Anxiety and Depression Scale Cognitive Bias Scale Vertigo Symptom Scale‐short form Adolescent Resilience Scale
Starting date	January 2022
Contact information	Reiko Tsunoda r.tsunoda@mejiro.ac.jp
Notes	We note that participants in this trial may have chronic dizziness, but not PPPD. However, as one outcome measure appears to specifically address PPPD (the Niigata PPPD Questionnaire) we have included this ongoing trial, as it may recruit people specifically with PPPD.

PPPD: persistent postural‐perceptual dizziness

## Differences between protocol and review

The protocol for this review stated that we would include a small number of specified non‐pharmacological interventions: talking therapies and vestibular rehabilitation. However, whilst preparing this review it became apparent that very few studies had been conducted in this field. We therefore expanded our list of relevant interventions to include any non‐pharmacological intervention that had been assessed for PPPD. 

We used the Trustworthiness Screening tool, developed by Cochrane Pregnancy and Childbirth. At the protocol stage we intended to exclude studies from the main analysis if any concerns were identified when using this tool. However, as we only identified one study for inclusion, and only one issue was identified (concerning randomisation), we did not use the tool to select studies for inclusion in the main analyses. 
